# Characterization of a Highly Virulent Noncytopathic Bovine Viral Diarrhea Virus 2b Isolate Detected in Cattle in Inner Mongolia, China

**DOI:** 10.1155/2023/3821435

**Published:** 2023-03-10

**Authors:** Chunxia Chai, Fan Zhang, Yun Diao, Yanyan Zhou, Shaowei Li, Hua Yin, Jinlong Zhang, Rui Niu, Wei Wang

**Affiliations:** ^1^State Key Laboratory of Reproductive Regulation and Breeding of Grassland Livestock, Inner Mongolia University, Hohhot 010030, China; ^2^Veterinary Research Institute, Inner Mongolia Academy of Agricultural & Animal Husbandry Sciences, Hohhot 010030, China

## Abstract

Bovine viral diarrhea virus (BVDV) infection causes subclinical to severe acute disease in cattle all over the world. Two genotypes of BVDV are recognized, BVDV1 and BVDV2. Currently, the subtypes of BVDV1b and BVDV2a are mainly circulating in China. Here, a newly noncytopathic (ncp) BVDV strain named BVDVNM21 was isolated and identified from cattle. We determined the complete genome sequence of BVDVNM21, phylogenetic analysis of 5′ untranslated region (5′UTR), N^pro^, and E2 gene, and complete genome showed the virus belongs to BVDV2b subtype and formed an independent clade within BVDV2b. Genome-wide sequence analysis showed that BVDVNM21 had high homology with SD1301 (98.10%) isolated from China. In the experimental infection study of guinea pigs and calves, they all developed similar clinical signs, including a pronounced and prolonged febrile response lasting more than 3 days and pronounced reduction in white blood cells (WBC) and platelets of more than 40%. Moreover, guinea pigs showed more acute infection characteristics after infection, and WBC decreased by more than 50% at 1 day postinfection (dpi), and they recovered in less than 3 days. The results indicated that the BVDVNM21 strain showed high virulence in calves. It was the first confirmed isolation of a highly pathogenic ncpBVDV2b in cattle, as well as the establishment of the BVDV-guinea pig model. This study may provide a basis for further research and control of the prevalence of BVDV2b in China.

## 1. Introduction

Bovine viral diarrhea virus (BVDV) is well known as one of the pathogen resulting in bovine respiratory disease complex (BRDC) throughout the world and causes major economic losses[1]. In addition, BVDV infections may also lead to diarrhea, respiratory disease, immunosuppression, and the generation of persistently infected (PI) animals, which are the main sources of infection [2]. BVDV belongs to the *Pestivirus* genus of the *Flaviviridae* family [3]. Currently, the International Committee on Taxonomy of Viruses (ICTV) recognizes four approved *Pestivirus* species: BVDV-1, BVDV-2, classical swine fever virus (CSFV), and border disease virus (BDV) [4].

BVDV is an enveloped, single positive-stranded RNA virus of about 12.3 kb. The viral genome comprises with a 5′- untranslated region (UTR), a single open reading frame (ORF), and 3′UTR. The polyprotein was cleaved by the host and virus proteases into four structural proteins (C, Erns, E1, and E2) and seven nonstructural proteins (N^pro^, p7, NS2-3, NS4A, NS4B, NS5A, and NS5B) depending on the virus biotype [5]. Usually, 5′UTR and N^pro^ regions have been used for phylogenetic analysis and genotyping of BVDV isolates [6, 7]. However, E2 protein also shows high variability and was frequently used for genotyping of *Pestivirus* [8]. Currently, 21 subtypes of BVDV1 (1a–1u) [9, 10] and four subtypes of BVDV2 (2a–2d) have been identified [11–13]. Furthermore, the reliable classification of virus subtypes is extremely useful for epidemiological investigations, enabling monitoring of *Pestivirus* species prevalence and distribution, and identification of emerging viruses [2].

In addition, two biotypes were classified as cytopathic (cp) or noncytopathic (ncp) depending upon the effect on cell culture [14]. The virulent virus causes acute illness, but still the low virulent virus may generate a significant secondary infection. However, episodes of severe hemorrhagic illness have been linked to BVDV2. When an antigenically closed cp-BVDV and an antigenically closed ncp-BVDV coinfect the same cattle, the PI calf, which sheds the virus throughout its life, is at the risk of developing mucosal disease (MD). It is critical for determining the pathogenicity of a newly discovered virus in animals and following the disease's epidemiology [15].

So far, many BVDV strains have been isolated in China. Two strains, cpBVDV2a/SD-06 [16] and cpBVDV2a/JZ05-1 [17], were isolated from cattle; other two strains, cpBVDV2a/HLJ-10 and ncpBVDV2a/SH28, isolated from commercial fetal bovine serum were reported [18]. Furthermore, the BVDV spread in China is basically 1b and 2a subtypes [13, 17–21]. In 2021, a cattle farm that had not been vaccinated with the BVDV vaccine developed a disease with cough, diarrhea, and asthma in the Inner Mongolia Autonomous Region. In order to determine the pathogen of the disease, we examined field samples from the pathological material obtained from sick animals. One ncpBVDV2b strain was isolated using MDBK cell cultures and identified by the virus neutralization test, reverse transcriptase-polymerase chain reaction (RT-PCR) method, and immunofluorescence assay (IFA). This work aims to detect the virulence and molecular characteristics of newly isolated BVDVNM21 strain and provides a basis for further research and control of the prevalence of BVDV2b in China.

## 2. Materials and Methods

### 2.1. Cells and Viral Strains

MDBK cells were used for virus isolation. The cells and culture in this study were detected by RT-PCR and IFA, and no BVDV was found.

The BVDVNM21 strain was isolated and stored in our laboratory. The BVDV SD1301 strain (BVDV2b, ncp biotype, moderate virulence, GenBank: KJ000672) was used as the positive control and was procured from our laboratory [22].

### 2.2. Virus Isolation

Nasal swab samples were collected from sick calves that are not vaccinated with the BVDV vaccine in the Inner Mongolia Autonomous Region of China with symptoms including fever, diarrhea, anorexia, emaciation, coughing. The nasal swab samples were inoculated into MDBK cells and cultured in DMEM (HyClone, USA) with horse serum (HyClone, USA) in 37°C, with 5% CO_2_ incubator for 5 days. The cytopathic effects (CPEs) were observed and recorded daily. If the CPE was not observed, the virus was detected by an IFA test using a BVDV2 type-specific monoclonal antibody (VMRD, USA) as described previously [23].

### 2.3. RT-PCR Amplification

Total RNA was extracted from the virus-infected MDBK cells using the QIAamp viral RNA mini kit (Qiagen, Germany) according to the manufacturer's instructions. Using RNA as a template, single-stranded cDNAs were generated by PrimeScript™RT reagent kit (Takara, Japan) according to the manufacturer's instructions. The primers were targeted for 5′UTR : F-5′-CATGCCCATAGTAGGAC-3′; R-5′-CCATGTGCCATGTACAG-3′.

### 2.4. Nucleotide Sequencing and Phylogenetic Analysis

In order to further confirm the identity of the isolate strain, the virus was submitted for complete genome sequencing, the whole genome sequence was submitted to the GenBank database (Accession Numbers: OP792028). Phylogenetic analyses were carried out by MEGA 7.0 with neighbor-joining (NJ). The bootstrap values were determined from 1000 replicates of the original data. DNASTAR software is used to analyze homologous comparisons with related genes of reference strains included in GenBank.

### 2.5. Animals and Infection

Nine two-month-old Mongolian calves were randomly divided into three groups and tested negative for BVDV, BHV-1, and BPIV-3. RT-PCR was used for the antigens of BVDV [10] and BPIV-3 [24], PCR was used for the antigens of BHV-1 [25], and an enzyme linked immunosorbent assay (ELISA) was used for the antibodies (IDEXX, USA). In the previous stage, the infection dose range was confirmed by [25, 26], and the infection dose was confirmed by a gradient infection experiment. Three calves were intranasally inoculated with 6 ml of cell-culture medium containing 10^7.0^ TCID_50_/ml of the BVDVNM21 strain, three calves were intranasally inoculated with 6 ml of 10^7.0^ TCID_50_/ml of the BVDV SD1301 strain as a positive control group, and the calves in the negative control group were inoculated with DMEM.

The guinea pig model has been considered a reliable tool by the Argentina government and has been proposed by the Veterinary MedicinesAmericas Committee for Veterinary Medicines (CAMEVET) as a laboratory model for the study of the efficacy of the BVDV vaccine [27]. Twelve guinea pigs weighing 250 g were purchased from SPF Beijing, Biotechnology Co., Ltd. (Beijing, China) and divided into three groups randomly. All animals were placed in standard conditions and had free access to food and water. Guinea pigs were injected intranasally with the BVDVNM21 strain or the BVDV SD1301 strain at 10^6.2^ TCID_50_; DMEM was used as a negative control.

All calves and guinea pigs will be followed for clinical signs, such as depression, cough, asthma, and other respiratory diseases, as previously mentioned (Wang et al.). Their rectal temperature will be taken three times a day at a regular time for 14 days. WBC and platelet counts were automatically detected by the IDEXX ProCyte Dx *∗* Hematology Analyzer (IDEXX, USA). Likewise, deep nasal swabs and EDTA-infused blood were also collected at the abovementioned time. Separated white blood cells (WBC) and nasal swabs were used for virus isolation to detect viremia and shedding.

### 2.6. Data Analysis

The data were presented as means, with error bars representing the mean's standard deviation (SD). The statistical significance was calculated using Student's *t*-test for one comparison and analysis of variance (ANOVA) for multiple comparisons. *p* values less than 0.05 are significant. GraphPad Prism 8™ (GraphPad Software, USA) was used for all the statistical analysis.

## 3. Results

### 3.1. Virus Isolation and Identification

**Figure 1:**
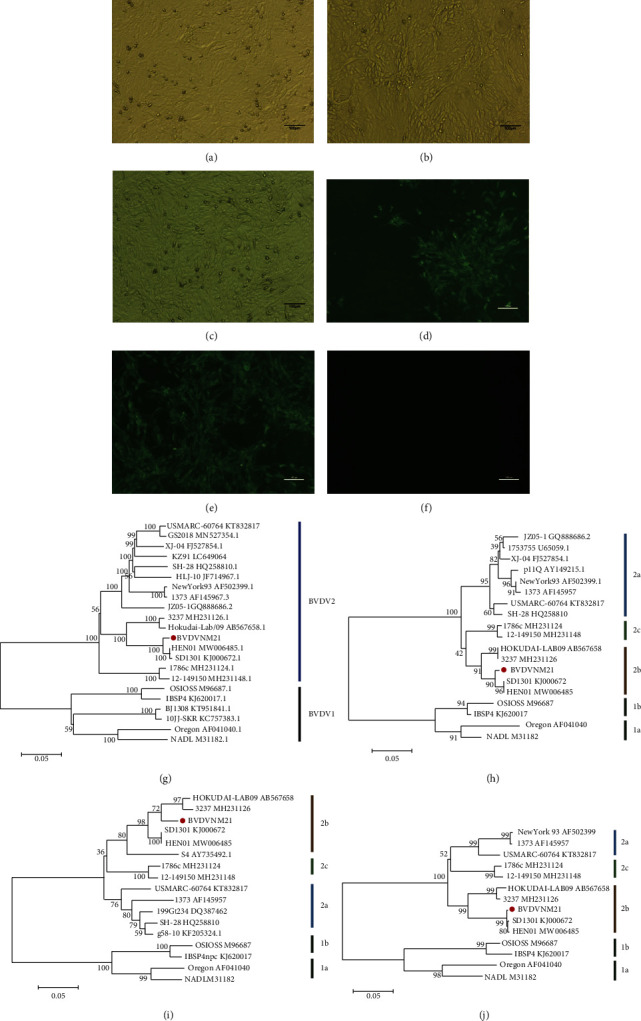
Cytopathic effects and phylogenetic analysis of the BVDVNM21 strain. (a) MDBK cells were infected with BVDVNM21 strain. (b) MDBK cells were infected with BVDVSD1301 strain (positive control). (c) Mock MDBK cells. MDBK cells were inoculated with (d) the BVDVNM21 and (e) the BVDVSD1301, and specific fluorescence was detected, but (f) uninfected cells did not show fluorescence. Phylogenetic tree analysis of (g) the complete sequence, (h) 5′UTR, (i) Npro, and E2 (j) was prepared using the neighbor-joining method by the software MEGA 7.0.

A virus was isolated from nasal swab samples by two blind passages on MDBK cells; the virus was of the ncp biotype. Specific fluorescent staining showed the appearance of granule distribution all over the cytoplasm in the infected MDBK cells when an IFA test was conducted using BVDV2 type-specific monoclonal antibodies (Figures 1(a)–1(f)).

### 3.2. Sequencing and Phylogenetic Analysis

The complete genome of the strain BVDV2NM21 was of 12,233 nucleotides in length contained a single ORF of 11,694 nt, 5′UTR of 335 nt, and 3′UTR of 203 nt. The identity of the nucleotide sequences shared with reference sequences is summarized in Table 1. It showed that the virus was related to the strains SD1301 (KJ000672) and HEN01 (MW006485) with a high homology of 98.10% and 97.88%, respectively.

Phylogenetic trees were constructed by comparing the complete sequence, 5′UTR, N^pro^ and E2 sequences of NM21 and other BVDV obtained from GenBank. The results showed that the NM21 strain was classified as the BVDV2b subtype and formed an independent clade within BVDV2b (Figures 1(g)–1(j)).

### 3.3. Experimental Infection

All calves infected with the BVDVNM21 strain developed clinical signs, including elevated rectal temperature, depression, leukopenia, thrombocytopenia, and nasal discharge. Two of the calves in the infected group showed significant aqueous diarrhea from 7 to 11 dpi, while the infected guinea pigs did not show obvious symptoms of diarrhea, and animals in the control group were normal.

**Figure 2:**
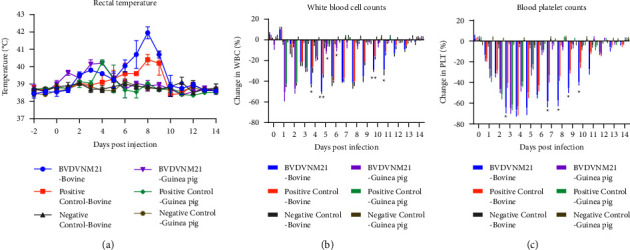
(a) The change in rectal temperatures, (b) the change in WBC, and (c) the change in blood platelet counts recorded within 14 days after infection. Values for each time point represent the average of animals. Significant differences are indicated with asterisks (^*∗*^*p* < 0.05 and ^*∗∗*^*p* < 0.01).

All three infected calves showed elevated rectal temperatures. Two of them presented biphasic pyrexia, with the first peak between 2 and 4 dpi and a maximum of 39.8°C; the other peak appeared at 8 dpi with a maximum temperature of 42.3°C. Similarly, guinea pigs also developed biphasic pyrexia and recovered quickly. The first peak appears at 1 dpi (39.6°C); the second peak appears at 4 dpi (40.2°C), while the positive control did not show biphasic pyrexia and the maximum temperature of 41.3°C in cattle. The negative control group had a body temperature within the normal range (Figure 2(a)).

In the infected calves group, the average WBC decline from 3 dpi to 8 dpi was more than 40% of the baseline value, and the peak value was 50.7% at 5 dpi. In the positive group of calves, the average WBC decline from 6 dpi to 8 dpi was more than 40% of the baseline value. Compared with the positive control, the average WBC decline in the infected group was significantly different at 4 dpi and 5 dpi. In contrast, values for uninfected calves ranged from -4.1% to 2.9% of baseline, within the normal range. Interestingly, the average WBC decline of infected guinea pigs was more than 40% of the baseline value only occurred at 1-2 dpi, and the peak value was about 51% at 1 dpi. However, there was no significant difference between the infected group and the positive control group of guinea pigs. The average WBC values in the control group ranged from -4.8% to 3.1% (Figure 2(b)).

Compared with the negative control group, the average platelet count of all calves decreased significantly from 3 dpi to 10 dpi (>40%). The maximum average platelet counts were decreased by 66.7% at 4 dpi. Compared with the positive group of calves, the average platelet count decrease degree was significantly different from 7 dpi to 10 dpi. The average platelet count of all guinea pigs declined from 2 dpi to 4 dpi by more than 40% of the baseline value; the peak value was 65.4% at 3 dpi, and no significant difference was observed in the positive control group (Figure 2(c)).

### 3.4. Viremia and Shedding

Nasal swabs from calves and guinea pigs were detected for viral shedding after infection. The results showed that calves and guinea pigs infected with BVDVNM21 had positive shedding from 2 dpi to 10 dpi. The virus isolated from blood samples of calves was positive for up to 12 days after inoculation, compared to 10 days in the positive control group of cattle, and a similar phenomenon occurs in guinea pigs (Table 2). Consequently, all infected animals developed viremia, and BVDV was isolated.

## 4. Discussion

In this study, a novel ncpBVDV2b strain named BVDVNM21 was isolated and identified in China. The whole genome and its pathogenesis in cattle and guinea pigs were determined, which will help better understand the epidemiology of BVDV2b in China and provide support for the disease's prevention and control in the cattle population.

Previous studies showed that the genotype of BVDV was associated with geography [28, 29]. The BVDV2a genotype was the dominant genotype in the North America. A number of BVDV2 isolates from Brazil and Argentina have been described in the literature [30, 31]. In contrast, BVDV2 appears to be relatively rare in Europe and Asia [32–34]. We also discovered that the BVDV2 was not reported in China prior to 2008 [13]. In 2009, when BVDV2a was first reported [16], it suggested that the BVDV2 was prevalent in China.

Phylogenetic analysis is useful for molecular epidemiological studies and vaccine research, in addition, tracing the origin of newly introduced viruses. The most conservative genes of 5′UTR, N^pro^, and E2 were an ideal choice for genotyping *Pestivirus* species [15, 28, 35, 36]. Results showed that the BVDVNM21 strain formed an independent clade within BVDV2b and had the highest identity with the strain SD1301 (98.10%) originated from China. It was reported that the virulence of BVDV2b strain SD1301 was moderate [22]. Some researchers searched for common features as virulence markers from the perspective of gene sequence, such as hypervirulent BVDV2 isolates containing an extra 16 amino acid peptide (-SSCPVPFDPSCHCNYF-) at C-terminal NS2 region [37] and a correlation between the presence of an uracil and a cytosine residue in positions 219 and 278 of the 5′UTR [38]. However, more studies have pointed out that there is no clear correlation between specific sequence motifs and virulence [39, 40]. Therefore, it is necessary to characterize virulence and pathogenicity in animals.

Some studies have shown that severe BVDV infection may be related to ncpBVDV2 strains [41]. In fact, BVDV2 also has weakly virulent strains [29]. Therefore, the clinical symptoms of BVDV acute infection are determined by the viral strain and immune status [42]. In addition, different ncpBVDV strains exhibit different virulence, even with the same genotype. Among the strains belonging to BVDV2, the NY-93 strain (2a) is virulent and the 98–124 strain (2b) is weak [43]. Moreover, KY1203, H0916, and HV03 with the same BVDV1a subtype showed low, high, and moderate virulence [26, 44], respectively.

According to observation in vivo, the clinical manifestations of infection with highly virulent BVDV2 strains include more severe fever for 3 days or more days (temperatures over 40°C up to 41.7°C) and a decrease of greater than 40% in circulating lymphocytes and platelets [45, 46]. In this study, calves and guinea pigs both presented biphasic pyrexia, with the second peak lasting for 4 days over 40°C, which has been reported in colostrum-deprived calves [45, 47]. All calves infected with the BVDVNM21 provoked a reduction in WBC counts and platelets greater than 40% for over 5 days, which was higher than that in the moderately virulent positive controls. Thus, the BVDVNM21 strain showed high virulence in calves. However, guinea pigs experienced transient leukopenia and thrombocytopenia less than 3 days, then quickly recovered, and positive control strain BVDV SD1301 also exhibited similar characteristics. We speculate that this phenomenon is caused by species differences, similar situations occurred in BALB/*c* mice infected with BVDV [48].

At present, with the rapid development of animal husbandry in China, we need to pay more attention to the popularity of a new highly virulent mutant of BVDV. The study of virulence variation is important to understanding the mechanisms behind pathology. The highly virulent BVDV can cause immunosuppression in calves, leading to secondary infection and a severe acute syndrome. The continuous emergence of new variants and strains of BVDV may hamper the efficacy of currently available diagnostic assays and vaccines [49]. Therefore, we need to make more efforts to prevent the emergence and spread of BVDV in order to eradicate this important virus from all over the world.

## Figures and Tables

**Table 1:** Nucleotide identity of NM21 with reference sequences.

Strain	Accession	Biotype	Year	Country	Nucleotide identity (%)			
					Complete	5′UTR	N^pro^	E2
SD1301	KJ000672	NCP	2012	China	98.10	91.67	95.83	99.64
HEN01	MW006485	NCP	2014	China	97.88	85.97	95.83	99.55
3237	MH231126	NCP	1990	USA	90.68	82.46	93.65	88.98
Hokudai-lab/09	AB567658	NCP	2010	Japan	89.84	81.03	91.87	87.90
JZ05-1	GQ888686	CP	2005	China	85.04	77.18	82.94	81.72
XJ-04	FJ527854	CP	2004	China	84.20	76.86	85.12	82.35
USMARC-60764	KT832817	NCP	2014	USA	84.17	80.85	84.52	81.90
12-149150	MH231148	NCP	2012	USA	84.00	83.61	83.73	81.36
SH-28	HQ258810	NCP	2009	China	83.99	78.15	82.34	80.82
1373	AF145967	NCP	1993	USA	83.38	75.96	82.94	79.66
New York 93	AF502399	NCP	1993	USA	83.37	76.35	82.94	80.38
1786c	MH231124	Unknown	1989	USA	81.74	80.42	83.53	82.17

**Table 2:** Viral isolation from blood samples or nasal swabs.

Group	Animals	Positive (%)							
		Baseline	Day 2	Day 4	Day 6	Day 8	Day 10	Day 12	Day 14
Cattle-nasal swabs (ns)	3	0	100	100	100	100	66.67	0	0
Cattle-blood samples (bs)	3	0	66.67	100	100	100	100	100	33.33
Positive control-cattle (ns)	3	0	33.33	100	100	66.67	0	0	0
Positive control-cattle (bs)	3	0	0	66.67	100	100	66.67	33.33	0
Guinea pigs-nasal swabs	4	0	50	100	75	50	25	0	0
Guinea pigs-blood samples	4	0	75	100	100	100	25	25	0
Positive control-Guinea pigs (ns)	4	0	50	100	100	50	0	0	0
Positive control-Guinea pigs (bs)	4	0	50	100	75	25	25	0	0
Negative control-cattle (ns/bs)	3/3	0	0	0	0	0	0	0	0
Negative control-Guinea pigs (ns/bs)	4/4	0	0	0	0	0	0	0	0

## Data Availability

The data used to support the findings of this study are included within the article.
